# A Case of Branch Retinal Artery Occlusion With Concomitant Intraocular and Extraocular Toxoplasmosis Lesions in a Japanese Man

**DOI:** 10.1155/crop/6654053

**Published:** 2025-06-02

**Authors:** Gen Kinari, Mizuki Tagami, Mami Tomita, Norihiko Misawa, Atsushi Sakai, Yusuke Haruna, Taro Shimono, Shigeru Honda

**Affiliations:** ^1^Department of Ophthalmology and Visual Sciences, Graduate School of Medicine, Osaka Metropolitan University, Osaka, Japan; ^2^Department of Diagnostic and Interventional Radiology, Graduate School of Medicine, Osaka Metropolitan University, Osaka, Japan

## Abstract

**Purpose:** The purpose of the study is to report a case of ocular toxoplasmosis with branch retinal artery occlusion (BRAO).

**Case:** The patient was a 36-year-old man from Okinawa, Japan, who was generally healthy and had no medical history. He was referred to our hospital with a complaint of sudden loss of vision in the left eye. Best corrected visual acuity was 0.1 in the left eye at the initial examination, and intraocular pressure was 21 mmHg. Anterior segment slit-lamp examination showed a few cells of the anterior segment and anterior vitreous. Fundus examination of the left eye showed retinal vasculitis of the middle and large retinal vessels with occlusion of retinal arterioles in the macular area and edema in all layers of the retina, with dense posterior vitreous cells and flare. Magnetic resonance imaging (MRI) showed a mass shadow at the posterior part of the left eyeball near the macula in the orbit. On laboratory examination, toxoplasma serum IgM and IgG were positive. Based on these results, he was diagnosed with BRAO with concomitant intraocular and extraocular toxoplasmosis. He was then treated with clindamycin, sulfamethoxazole–trimethoprim, and steroids. The inflammation disappeared quickly, and visual acuity improved to 0.6. The inflammation had not flared up even 3 months after the initial visit with decreasing serum toxoplasma serum IgG levels, and the posterior eyeball shadow on the MRI disappeared.

**Conclusion:** A case of BRAO with uveitis with concomitant intraocular and extraocular toxoplasmosis lesions was presented. In cases of unilateral retinal vasculitis with orbital lesions, concomitant intraocular and extraocular toxoplasmosis should also be considered.

## 1. Introduction

Ocular toxoplasmosis is a rare disease, but it has been reported as one of the leading causes of uveitis. Although studies vary, toxoplasmosis generally accounts for approximately 1%–30% of uveitis cases [1, 2].

Toxoplasma is an intracellular parasitic protozoan parasite whose end host is the cat, and humans are intermediate hosts. It is transmitted to humans through oocysts in feline feces or by eating undercooked meat from cattle, pigs, chickens, or other intermediate hosts infected with toxoplasma [3].

Ocular toxoplasmosis has been reported to cause a variety of ocular fundus diseases. Atypical symptoms have been reported in the literature, including optic neuropathy, angiogenesis, retinal vascular occlusion, epiretinal membrane, and retinal detachment [4]. A case of branch retinal artery occlusion (BRAO) caused by toxoplasma in a young man with no previous history of the disease is reported.

## 2. Case

**Figure 1:**
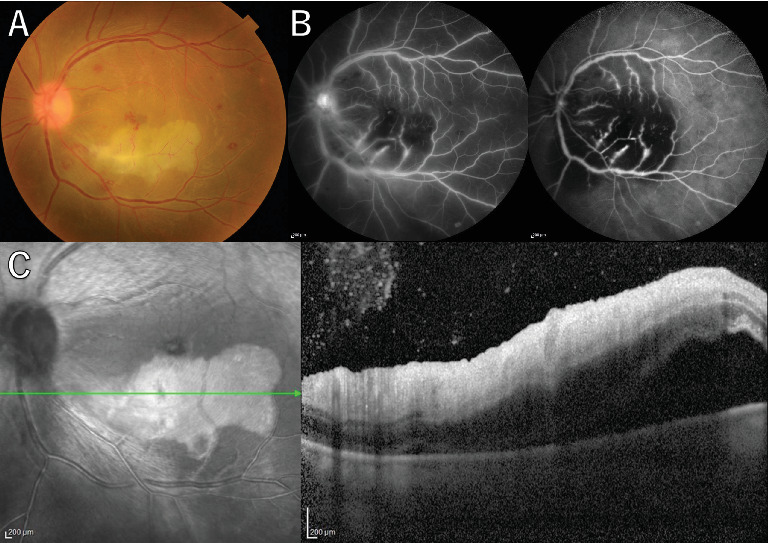
(A) Fundus photograph shows redness and swelling of the optic disc and yellowish-white retinal edematous lesions with branch retinal artery occlusion with retinal vasculitis in the left eye at the first visit. (B) Fluorescein angiography (FA) shows leakage due to vasculitis, and areas of macular nonperfusion were observed. (C) Optical coherence tomography (OCT) shows prominent hyperintense lesions in the central inner retina surrounding the occluded artery.

**Figure 2:**
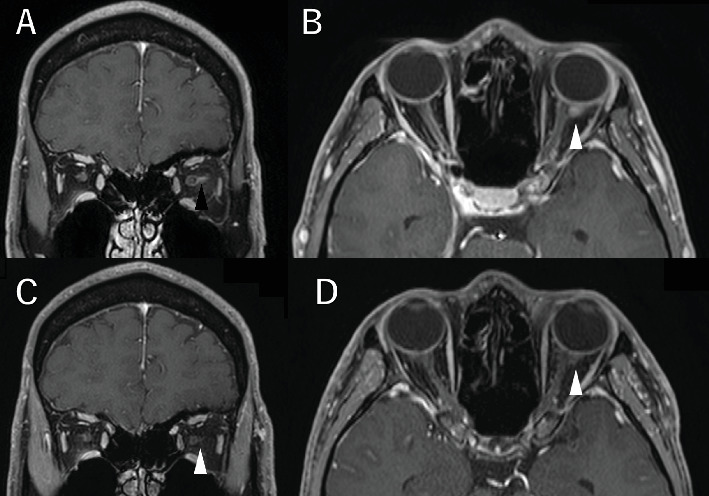
Magnetic resonance imaging (MRI) shows a mass shadow at the posterior part of the left eyeball near the macula in the orbit at the first visit: (A) T1 axial; (B) T1 coronal. On MRI 9 months later, the contrast effect of the mass shadow in the posterior part of the left eyeball has diminished. White arrowhead: orbital nodular lesion of orbital toxoplasmosis: (C) T1 axial; (D) T1 coronal with gadolinium-enhanced.

**Figure 3:**
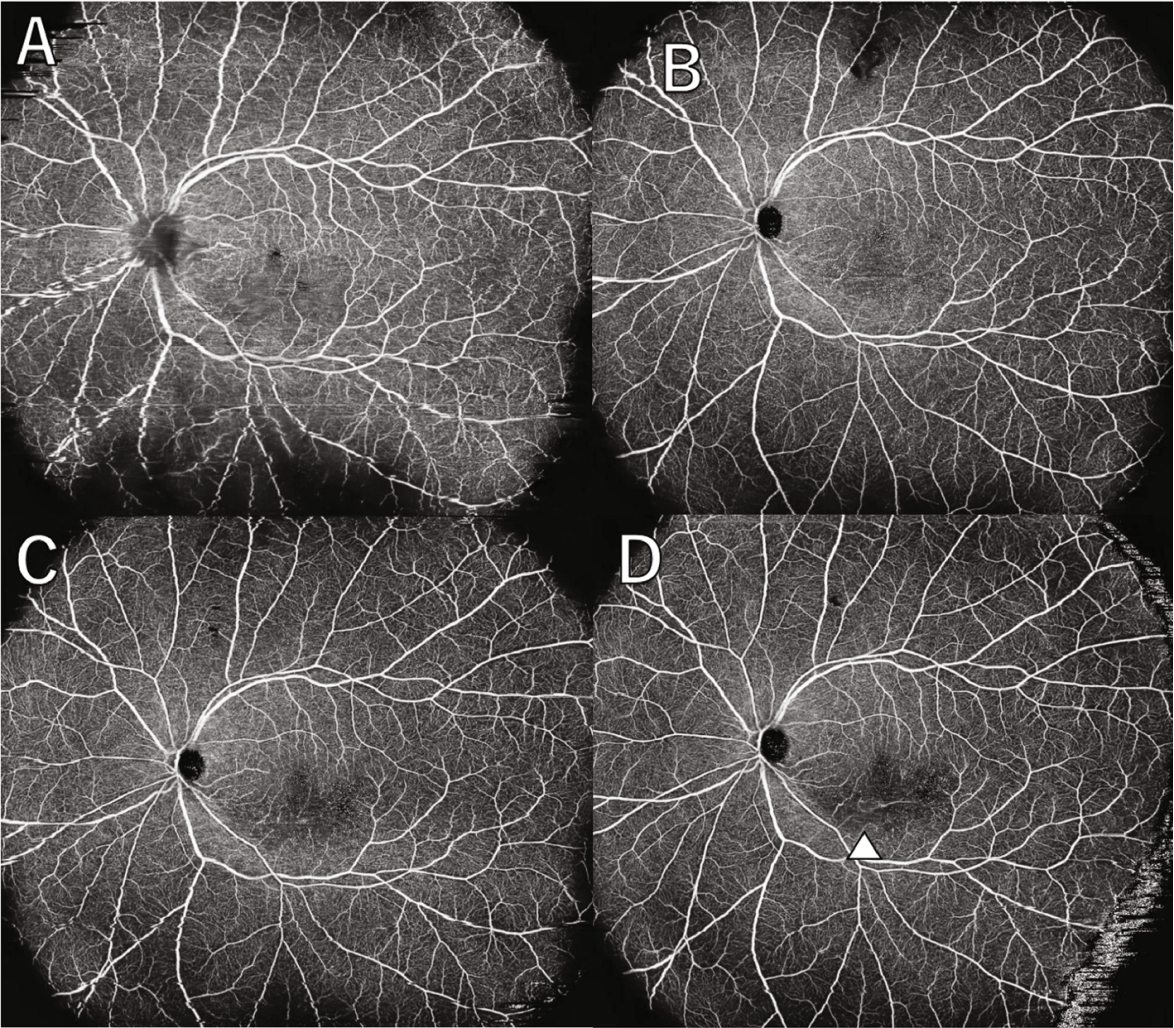
En-face ultra-widefield optical coherence tomography angiography (OCTA) images of the left eye: (A) the first visit; (B) 1 month after surgery; (C) 3 months later; (D) 9 months later (red arrowhead: optic nerve papillary swelling; white arrow: poor circulation). At the time of the first visit, the acute findings were equivocal with papilledema and retinal edema, but over the course of 9 months, the decrease in blood flow over time is clearly demonstrated.

**Figure 4:**
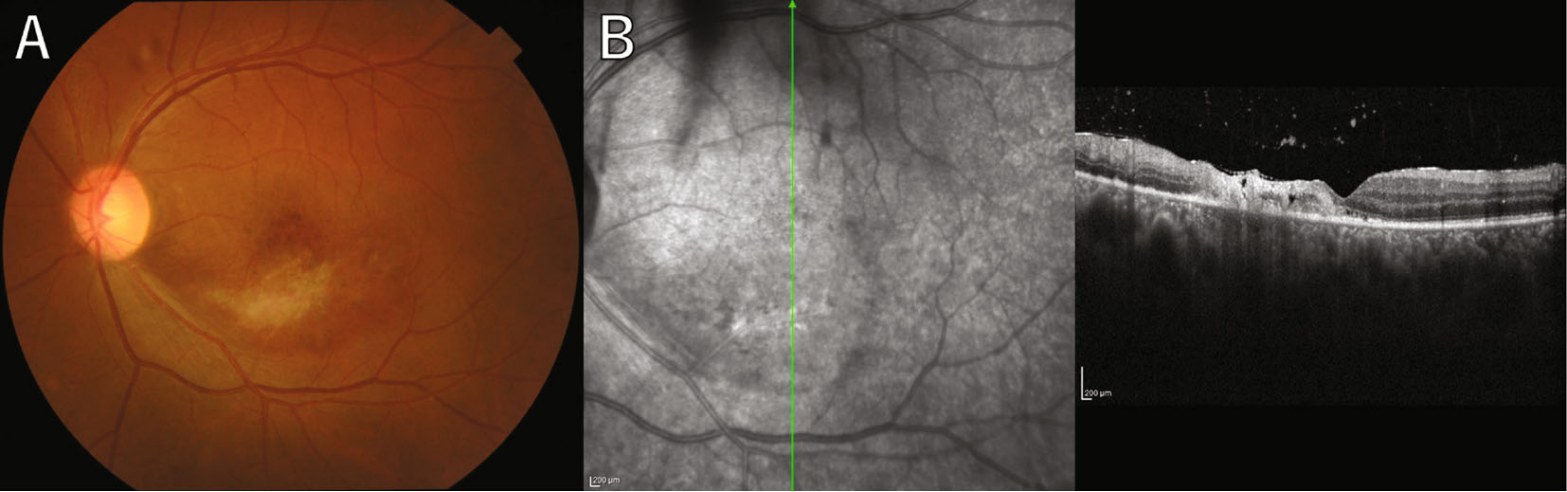
(A) Fundus photograph shows an almost normal color of the optic disc and retinal atrophic change with a sheathing artery near the macula in the left eye at the final visit. (B) Optical coherence tomography (OCT) shows a thinner inner retina on the macula surrounding hyperreflective lesions.

A 36-year-old man with a chief complaint of a sudden loss of vision in the left eye that began 1 day earlier was referred to our department after a visit to his previous doctor who found BRAO. The patient had been in good health and had no underlying disease. The left eye's best corrected visual acuity was 0.1. No abnormalities were found in the light reflex, with no relative afferent pupillary defect (RAPD). There was no inflammation in the anterior segment of the eye. Fundus examination of the left eye showed retinal vasculitis of the middle and large retinal vessels with occlusion of retinal arterioles in the macular area and edema in all layers of the retina (Figure 1). Goldmann perimetry (GP) showed decreased sensitivity and central darkening consistent with a vascular occlusion area; fluorescein angiography (FA) showed delayed filling in the inferior part of the left eye arcade, and leakage from the optic disc in the early phase and hypofluorescence in the late phase (Figure 1) were also observed. Ocular coherence tomography (OCT) showed intraretinal edema, serous retinal detachment, and vigorous vitreous cells in the left eye (Figure 1). There was no leak in the peripheral vessels. Based on these findings, the patient was diagnosed with left BRAO. Since the patient was a young man with no medical history, uveitis was suspected, and he was hospitalized for close examination and treatment after collecting anterior chamber fluid for comprehensive multiplex real-time polymerase chain reaction (PCR) testing by previous reported methods [5–7]. The anterior chamber fluid was negative for comprehensive infections. Prednisolone 125 mg and prostaglandin E1 as a vasodilator were administered intravenously for optic papillitis, and the patient was carefully watched for worsening inflammation. After betamethasone eye drops were started, vitreous inflammation worsened, and inflammation in the anterior chamber appeared. Anterior segment slit photography showed posterior corneal deposits and anterior chamber cells, and steroid administration was discontinued. Suspecting infectious retinitis, gadolinium-enhanced magnetic resonance imaging (MRI) of the orbital region was performed and showed a mass shadow at the posterior part of the left eyeball in the orbit (Figure 2). In addition, both toxoplasma serum IgM and IgG were positive, suggesting current infection (Table 1). Then, treatment for ocular toxoplasmosis was started with clindamycin 2400 mg and sulfamethoxazole–trimethoprim 3840 mg for 8 weeks. The intravitreal inflammation gradually improved, and the patient was discharged from the hospital. The retinal white matter lesions disappeared after discharge, and OCT and en-face ultra-widefield optical coherence tomography angiography (OCTA) showed thinning of the vascular occlusions compared to at the onset of the disease (Figures 3, 3, 3, 3, 4, 4, and 4). The final visual acuity improved to LV (0.6), and the orbital lesion in MRI showed that the contrast effect of the mass shadow in the posterior part of the left eyeball has diminished (Figure 2) after 10 months later.

The patient has been under observation with decreasing toxoplasma serum IgG antibody levels for the past 6 months without any worsening.

## 3. Discussion

A case of BRAO with uveitis accompanied by concomitant intraocular and extraocular toxoplasmosis lesions, in unilateral retinal vasculitis with orbital lesions, was described. This is the first case of ocular toxoplasmosis inside and outside the orbit with BRAO and examined using multimodal imaging.

In general, the diagnostic standard for uveitis of unknown origin is multiplex PCR, an established method for excluding an infectious origin, as reported by Sugita et al. and Nakano et al. [5, 7] In the present case, the results of two anterior chamber aqueous humor PCRs were both negative, and the positive results for toxoplasma IgM and IgG led to the diagnosis of ocular toxoplasmosis. The sensitivity of real-time PCR of intraocular fluid in protozoan-infected uveitis has been reported to be 57% [8]. In particular, the sensitivity and specificity for ocular toxoplasmosis have been reported to be low [9]. Although the sensitivity is low, it is inferred that PCR sensitivity may be higher in the vitreous, which is closer to the lesion, given that toxoplasma is a protozoan. In addition, epidemiological studies in Japan have reported that the infection rate of *Toxoplasma gondii* is high in animal hosts in the southwest islands of Japan, especially Okinawa, and although accurate national data do not exist, it is thought that work history and residential history are also important in cases of ocular toxoplasmosis [10]. Of course, there is a possibility of mother-to-child transmission, and it is unclear how a healthy young man who was not a compromised host developed the disease at this time. We suspect that the infection may have been acquired, but we are not certain.

It has been reported that BRAO occurred in 7% of ocular toxoplasmosis cases [11]. The cause was mentioned as direct compression of arteries (retinal vessels) or adjacent active retinitis lesions by inflammatory foci, vasoconstriction, and increased blood viscosity [11]. These speculations are supported by prior reports of retinal vasculitis and vascular occlusion in ocular toxoplasmosis [3, 12–14]. Furthermore, based on the results of experimental verification using monkeys, we speculated that the pathology was consistent[15]. As a limitation, in this case, *Toxoplasma gondii* mRNA was not detected in the intraocular fluid, so it was not a definitive diagnosis, but rather a diagnosis of exclusion. In the present case, the lesion was located in the posterior aspect of the eye near the optic nerve, which showed a contrast effect on orbital MRI, and inflammatory spillover from there may have caused the BRAO.

Reports of MRI findings in ocular toxoplasmosis are rare, but there are reports of circumferential contrast effects along the optic nerve [16]. There have been several reports of retinal choroiditis after steroid administration for optic nerve inflammation [17].

In the present case, as well, the worsening of inflammation after steroid treatment led us to consider infectious retinitis. Therefore, when administering steroids to young patients with optic nerve inflammation without a history of previous treatment, the fundus should be carefully monitored for the possibility of infectious lesions.

The present case also suggested, in particular, that, especially with regard to toxoplasmosis, a comprehensive diagnosis of infection by multiplex PCR is not all powerful, and a detailed and accurate medical history and key imaging evaluations are what formed the basis of the ocular toxoplasmosis diagnosis.

In conclusion, BRAO with uveitis with concomitant intraocular and extraocular toxoplasmosis lesions was described. In unilateral retinal vasculitis with an orbital lesion, concomitant intraocular and extraocular toxoplasmosis should be considered.

## Figures and Tables

**Table 1:** Pretreatment blood data for hematology and serology: toxoplasma IgG and IgM were positive.

**Hematology**	**Result**	**Reference range**	**Unit**	**Serology**	**Result**	**Reference range**	**Unit**
WBC	5900	3300–8600	*μ*L	CRP	0.01	0–0.14	mg/dL
RBC	456 × 10	435–555	103/*μ*L	IgG	991	861–1747	mg/dL
Hb	12.2	13.7–16.8	g/dL	IgA	197	93–393	mg/dL
Ht	37.6	40.7–50.1	%	IgM	57	36–245	mg/dL
Plt	32.4 × 10	158–348	103/*μ*L	RF	2<	0–15.0	IU/mL
PT sec	11.1	9.8–12.1	s	HTLV-I ab	—	0–1.0	—
PT-INR	0.96	0.90–1.14	—	HPV-1 IgM	—	0–0.80	—
				VZV IgM	—	0–0.80	—
Biochemistry				**Toxoplasma IgM**	**1.2**	**0–0.80**	—
AST	22	13–30	U/L	**Toxoplasma IgG**	**134**	**0–6.0**	—
ALT	29	10–42	U/L	HIV	**—**		—
ChE	293	208–311	U/L	TB IFN-*γ*	**—**		—
UA	5.4	2.8–7.8	mg/dL	STS	**—**		—
BUN	14	−20	mg/dL	TPHA	**—**		—
Cre	0.88	0.65–1.07	mg/dL				
Na	141	138–145	mmol/L				
K	4.5	3.6–4.8	mmol/L				
Cl	104	101–108	mmol/L				

*Note:* Bold values indicate clinically significant findings.

## Data Availability

The data that support the findings of this study are available from the corresponding author upon reasonable request.

## Nomenclature

MRI: magnetic resonance imaging
BRAO: branch retinal artery occlusion
OCT: ocular coherence tomography
RAPD: relative afferent pupillary defect
GP: Goldmann perimetry
FA: fluorescein angiography
PCR: polymerase chain reaction
